# Incubation of craving: a Bayesian account

**DOI:** 10.1038/s41386-018-0108-7

**Published:** 2018-06-02

**Authors:** Xiaosi Gu

**Affiliations:** 1 School of Behavioral and Brain Sciences, The University of Texas at Dallas, Dallas, TX USA; 2 Department of Psychiatry, Icahn School of Medicine at Mount Sinai, New York, NY USA

In 1986, Gawin and Kleber observed that cocaine craving increased over time during early abstinence in humans [1]. It was not until 15 years later did Grimm and colleagues provide the first laboratory-based neuroscience evidence supporting the initial finding from Gawin and Kleber using cocaine-dependent rats and coin the term “*incubation of craving*” [2]. Since then, cellular and molecular mechanisms mediating incubation of craving for various addictive substances have been extensively studied in rodents (see [3–5] for reviews); yet in humans, such studies are still rare (see [6–9] for a few examples). Furthermore, current findings on the incubation of craving do not fully converge [5]. For example, while some studies suggest that craving steadily increases during early abstinence [2, 6, 10, 11], others show that beyond a certain time point, craving may decrease during a later stage of abstinence [7, 12, 13]. Difference in the measurement of craving might be an important contributor to the inconsistency in these findings—human studies have used self-reports of subjective craving [6, 8, 9] and electroencephalogram (EEG) signals [7] as indices for craving, while rat studies typically use lever press for substances as a measurement [3, 4, 13]. Furthermore, the handful of studies in humans have not yet been able to provide a mechanistic account for the incubation of craving in patients. Thus, it remains unknown *how* the human brain *computes* the incubation of craving at the algorithmic level.

Previously, we proposed a Bayesian observer model of craving, which considers craving as a Bayesian belief—about the physiological states of the body [14]. This model is grounded in the Bayesian brain framework, which suggests that the brain updates its beliefs about the world by combining prior knowledge and observations, based on the Bayes rule:

$${\rm{Posterior}}\;{\rm{belief}} = \frac{{{\rm{sensory}}\;{\rm{evidence}} \times {\rm{prior}}\;{\rm{belief}}}}{{{\rm{marginal}}\;{\rm{likelihood}}}}$$

Here, I demonstrate that incubation of craving can be simulated using the same Bayesian framework. As shown in Fig. 1a, at time point t1, the prior belief is associated with low craving as the agent inherently expects the desired drug to be delivered, which would reduce physical discomfort. The likelihood is the sensory evidence—the observation of the agent’s bodily states (e.g., increased heart rate, sweating, higher blood pressure) associated with the lack of drugs. Initially, this likelihood is on the right side of the *x-* axis because the level of physical discomfort is high due to the lack of drugs. The updated posterior falls in between the prior and the likelihood and represents an updated belief about the physiological states of the body.

**Fig. 1:**
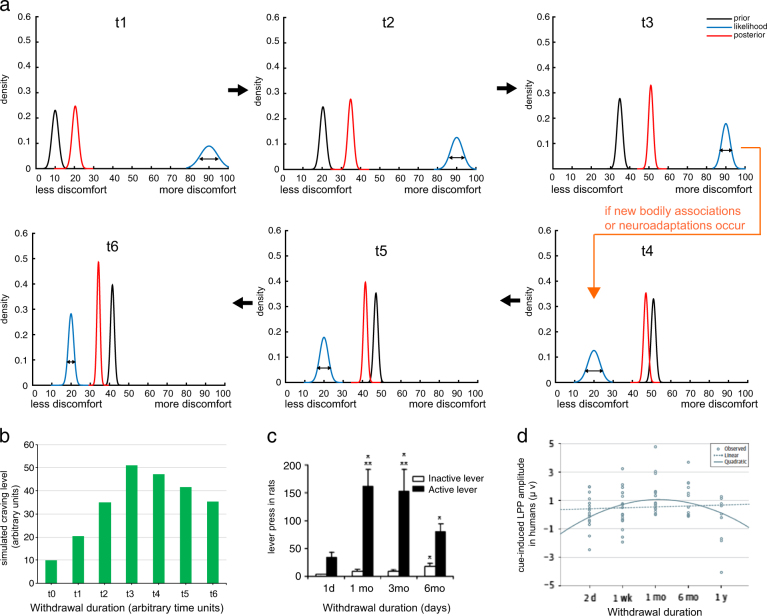
A Bayesian account for the incubation of craving effect. **a** Simulated Bayesian updating of craving over time during abstinence. Black curves represent the prior belief (e.g., based on experience), blue represents likelihood or sensory evidence (e.g. actual bodily states associated with the absence of drugs), and red curves are the posterior belief (as a result of prior and likelihood). Notice that the prior in each panel (excluding t1) is the posterior in the preceding panel. Early abstinence (upper panels t1–t3): posterior craving steadily increases. Late abstinence (lower panels t4–t6): posterior craving gradually decreases if there is a major shift in likelihood (e.g., due to forming new associations between bodily states and the lack of drugs or neuroadaptation). Note that in order for the posterior craving to decline, external factors will have to take effect so that the likelihood function shifts to the left end of the *x*-axis. However, it is also possible that neither new drug–body associations are formed nor neuroadaptations (e.g. recovery of D1 receptors) occur. In this case, the likelihood could remain on the right side of the *x*-axis (as in t1–t3) and as such, craving may persist, rather than decline, during late abstinence. **b** A bar graph representing the posterior craving levels simulated in (**a**). Notice the early increase is faster than the late decrease in craving, which mimics the craving patterns (shown in **c**, **d**). **c** Craving level (indexed by lever press) in cocaine-addicted rats increases during early abstinence but decreases during late abstinence. Adapted from [13]. **d** In human participants addicted to cocaine, an inverted U-shaped relationship was found between length of abstinence and cue-induced late positive potential (LPP) responses. Adapted from [7]

Two factors may contribute to the increase in craving during early abstinence in Fig. 1a (t1–t3). The first is the moving prior due to Bayes rule—“*yesterday’s posterior is today’s prior*”. For example, posterior from t1 becomes the prior at t2 and so on. Because the posterior belief is contingent upon both the prior and the likelihood, as long as the likelihood is to the right of the prior, the posterior will be moved towards the right-hand side of the *x*-axis (i.e., more discomfort) and interpreted as more craving, as is the case in early abstinence (Fig. 1a; t1–t3). A second factor contributing to incubation of craving during early abstinence is the greater precision  or peakiness of the likelihood (e.g., comparing the widths of blue curves in t1–t3), as the agent accumulates more evidence (e.g., the observation of no drug leading to more physical discomfort becomes more certain). As a result, the posterior belief is drawn even further away from the prior and towards the right side of the *x*-axis (i.e. more craving).

But what might contribute to the decline in craving in late abstinence as reported in [7, 13]? Such change cannot happen naturally within the current setup of the Bayesian model, if the likelihood stays the same. Instead, in order for the posterior craving to move back to the left side of the *x*-axis in Fig. 1a (t4–t6), external factors will have to take effect so that the observation of ‘no drug’ will no longer be associated with heightened bodily states (i.e. likelihood jumps to the left side of the *x*-axis). There are a few possibilities. First, the individual could override the old memory of drug–body associations with a new association that the lack of drugs may not necessarily lead to unpleasant bodily sensations. This has been proven plausible in humans, for instance, using cognitive therapy [16] or mindfulness techniques [17]. Second, certain neuroadaptive processes could take place and subserve the reduction in craving during late abstinence. For example, it has been shown that reduced dopamine D1 receptors during early abstinence could recover later when abstinence continues [15]. As such, the posterior belief can be moved back toward the left of the *x*-axis(i.e., less craving) during a later stage of abstinence (Fig. 1a, panels t4–t6). Additionally, similar to the early abstinence stage, tightening of the likelihood function (due to less uncertainty of the sensory evidence) could also occur and further contribute to the reduction of posterior craving. However, it is also possible that neither new drug–body associations are formed nor neuroadaptations (e.g., recovery of D1) occur. In this case, the likelihood could remain on the right side of the *x*-axis (as in Fig. 1a, t1–t3) and as such, craving may persist, rather than decline, during late abstinence. This could potentially account for the heterogeneity in craving and relapse profiles among addicted individuals.

 The proposed Bayesian model offers several testable predictions. First, this model predicts that the decline in late abstinence is slower than the speed of initial increase, as seen in Fig. 1c, d. This is because the prior belief (e.g., at t4) has already become highly precise/peaky in late abstinence. Thus, even if the likelihood moves to the left side of the *x*-axis later on, the influence of this new likelihood on the posterior belief is moderate. Second, this model also predicts that there could exist a variety of phenotypes in the incubation process due to individual differences in priors and how individuals accumulate sensory evidence. For example, craving in individuals with peakier likelihood functions (i.e., less uncertainty about the bodily effects of no drugs) would incubate faster than those with flatter likelihood distributions. Individuals with stronger baseline craving (i.e., prior is more toward the right side of the *x*-axis in Fig. 1a) will also reach the maximum level of craving faster, potentially relapse sooner. Third, the model predicts that the decline in craving may not happen for all individuals. If the likelihood does not change as shown in Fig. 1a (e.g., due to one’s inability to form new bodily states), craving will reach a stable high level and persist, as seen in some individuals. Last, strategies that remove drug-related memories could pontetially be effective in reducing craving, as these events can make the likelihood weaker or less precise, and thus prevent or delay the progression of craving.

Several important issues remain to be examined in this area of research. In particular, we need more empirical data from humans, especially longitudinal and neural data associted with the incubation of craving. Importantly, treatment studies focused on incubation of craving are almost non-existent. Such work is urgently needed to test the predictions offered by the proposed neurocomputatioinal model, which would advance our understanding of the mechanism of incubation of craving, and eventually, inform relapse prevention and successful recovery.

## Electronic supplementary material

Incubation of Craving

Incubation of Craving
